# Vigilin interacts with signal peptide peptidase

**DOI:** 10.1186/1477-5956-10-33

**Published:** 2012-05-18

**Authors:** Stephen Hsueh-Jeng Lu, Amy Hye Won Jeon, Gerold Schmitt-Ulms, Seema Qamar, Roger Dodd, Beth McDonald, Yi Li, William Meadows, Katie Cox, Christopher Bohm, Fusheng Chen, Paul Fraser, Peter St George-Hyslop

**Affiliations:** 1 Cambridge Institute for Medical Research, Department of Clinical Neurosciences, University of Cambridge, Addenbrooke's Hospital, Hills Road, England, Cambridge, CB2 0XY, United Kingdom; 2 Tanz Centre for Research in Neurodegenerative Diseases, and Departments of Medicine, Laboratory Medicine and Pathobiology, and Medical Biophysics, University of Toronto, 6 Queen's Park Crescent West, Toronto, ON, M5S 3H2, Canada

**Keywords:** Signal peptide peptidase, Vigilin, Biochemistry, Intramembrane-cleaving aspartyl protease, Non-proteolytic function, Interactome

## Abstract

**Background:**

Signal peptide peptidase (SPP), a member of the presenilin-like intra-membrane cleaving aspartyl protease family, migrates on Blue Native (BN) gels as 100 kDa, 200 kDa and 450 kDa species. SPP has recently been implicated in other non-proteolytic functions such as retro-translocation of MHC Class I molecules and binding of misfolded proteins in the endoplasmic reticulum (ER). These high molecular weight SPP complexes might contain additional proteins that regulate the proteolytic activity of SPP or support its non-catalytic functions.

**Results:**

In this study, an unbiased iTRAQ-labeling mass spectrometry approach was used to identify SPP-interacting proteins. We found that vigilin, a ubiquitous multi-KH domain containing cytoplasmic protein involved in RNA binding and protein translation control, selectively enriched with SPP. Vigilin interacted with SPP and both proteins co-localized in restricted intracellular domains near the ER, biochemically co-fractionated and were part of the same 450 kDa complex on BN gels. However, vigilin does not alter the protease activity of SPP, suggesting that the SPP-vigilin interaction might be involved in the non-proteolytic functions of SPP.

**Conclusions:**

We have identified and validated vigilin as a novel interacting partner of SPP that could play an important role in the non-proteolytic functions of SPP. This data adds further weight to the idea that intramembrane-cleaving aspartyl proteases, such as presenilin and SPPs, could have other functions besides the proteolysis of short membrane stubs.

## Background

Signal peptide peptidase (SPP) [1] is a member of the intramembrane cleaving aspartyl protease family that also includes the presenilins and SPP-like proteases. Members of this family share characteristic YD, GxGD and PAL motifs [2]. However, the presenilins cleave Type I transmembrane proteins [3], while the SPP-like proteases cleave Type II transmembrane proteins [1]. Amongst the SPP-like proteases, the ER-resident SPP is thought to cleave the membrane-bound stubs of some secreted proteins following proteolysis of the signal peptide by the signal peptidase [1,4], and in doing so presumably releases the stubs from the ER membrane. SPP may also have additional activities in protein control in the ER. For example, SPP is required for the dislocation or retro-translocation of MHC Class I molecules in the presence of the human cytomegalovirus (hCMV) US2 protein [5]. Others have reported that both catalytically inactive and wild-type SPP may bind and stabilize misfolded membrane proteins [6,7].

**Figure 1:**
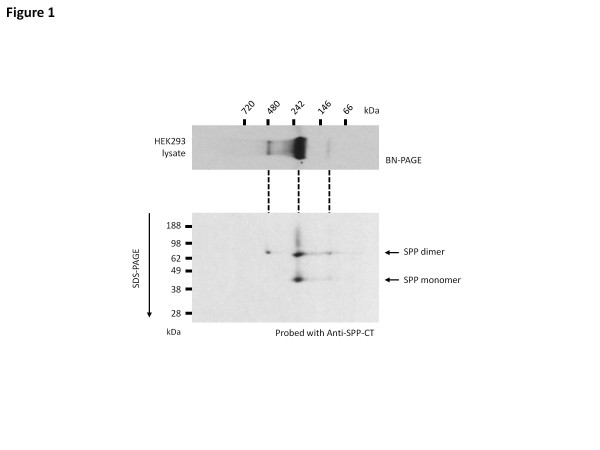
**SPP exists in higher molecular weight complexes.***Top panel*, HEK293 lysate solubilized in 0.5% DDM and resolved on 16% Bis-Tris Blue Native-PAGE gels, revealing SPP-containing complexes at around 450 kDa, 200 kDa and 100 kDa. *Lower panel*, lysates resolved on the BN-PAGE were then resolved on the second dimension/SDS-PAGE. SDS-stable SPP dimer can be found in all three high molecular weight bands suggesting that the 100 kDa band observed in the first dimension is a SDS-stable SPP dimer.

Preliminary biochemical and biophysical studies by this and other groups [7] have revealed that the SPP-like proteases in general, and SPP in particular, exist in cell lysates as higher molecular mass species than would be predicted from the calculated molecular weight of their respective monomeric polypeptides (Figure 1). One potential explanation for this observation is that the SPP proteins might exist as dimers [8,9] or higher order assemblies. However, this observation also raises the possibility that there may be ancillary proteins which, although not required for SPP proteolytic activity, may nevertheless modulate its function. We therefore set out to determine whether the high molecular weight SPP complexes might contain other proteins, and if so, whether these other proteins modulated the SPP protease activity function of the SPPs. Using an unbiased mass spectrometry (MS) based approach, vigilin was identified as a candidate SPP interacting protein. Reciprocal co-immunoprecipitations confirmed the interaction between SPP and vigilin. Additionally, SPP and vigilin co-localized in restricted cellular domains, co-fractionated biochemically and co-migrated as a single band on BN gels.

## Results

### Isolation of endogenous SPP complexes

**Figure 2:**
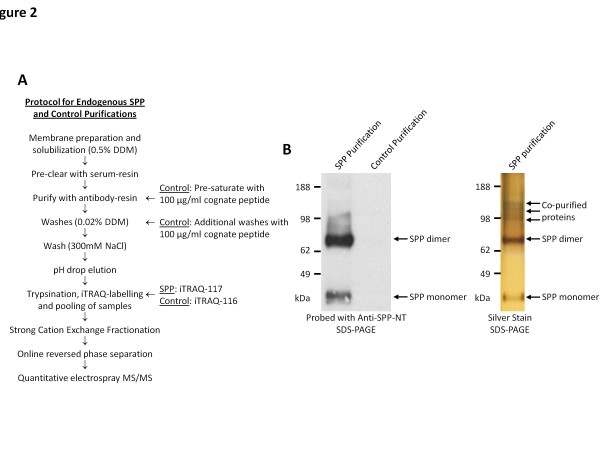
**Purification of SPP containing complexes.** (**A**) Schematic showing the protocol for the purification and iTRAQ-based mass spectrometry analysis of the SPP interacting proteins. (**B**) Purifications were analysed and compared by Western blot with anti-SPP antibody (*left panel*), demonstrating that the control purification protocol prevented SPP from being purified, and silver staining (*right panel*), showing the presence of several co-purified proteins that may represent novel SPP interacting proteins.

**Figure 3:**
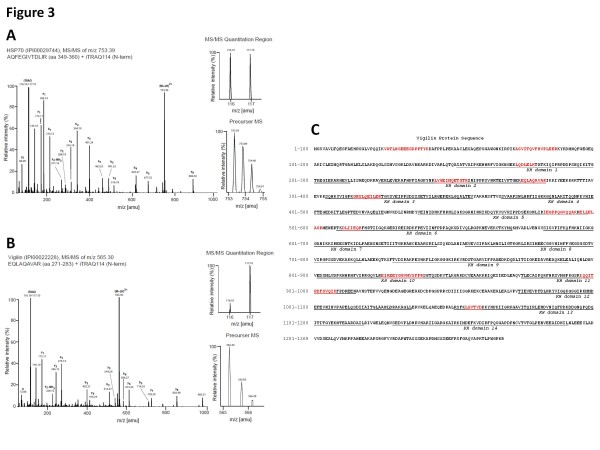
**Representative CID spectra documenting specific co-enrichment of vigilin with SPP and non-specific co-purification of HSP70.** Parallel co-purification with pre-saturated or naive anti-SPP antibody was followed by iTRAQ-based quantitation and ESI-MS/MS analyses. iTRAQ labeling reactions were setup with the iTRAQ116 label as the negative control, and iTRAQ117 label as peptides derived from the SPP-specific purification. (**A**) CID spectrum assigned to tryptic peptide AQFEGIVTDLIR which contributed to the identification of HSP70. iTRAQ signature mass peak ratios documented that this peptide was contributed equally by negative control and specific IP eluates. (**B**) Representative CID spectrum assigned to vigilin-derived peptide EQLAQAVAR which exhibited a strongly skewed iTRAQ ratio, demonstrating that this peptide was primarily contributed by the specific sample. Graphs on the side depict expanded views of the isotopic envelope of the respective precursor masses and the iTRAQ signature mass peaks. (**C**) Human vigilin amino acid sequence (GenBank: NP_005327.1) with peptides identified by MS/MS are in red and the 14 KH domains are underlined.

SPP and its potential interacting partners were isolated from native HEK293 cells using a single-step affinity purification protocol (for a schematic of the protocol see Figure 2A). Specifically, microsomal membrane isolates were solubilized in 0.5% N-Dodecyl-β-D-maltoside (DDM) and subjected to anti-SPP affinity purification using anti-SPP-CT antibody, which recognizes the C-terminal amino acids 358–377 of SPP. In order to generate the negative control with which to identify and exclude non-specific binders, the capture antibody was pre-blocked with cognate peptide in the control purification, and the cognate peptide was further included during the subsequent affinity matrix washing step. The purified SPP proteins were resolved by SDS-PAGE and visualized by Western blotting and silver staining (Figure 2B). In eluates obtained following SPP-specific capture, SPP-immunoreactive bands were observed at 42 kDa for monomeric SPP (predicted to be 47 kDa) and at 70 kDa, interpreted to be SDS-stable dimers. The discrepancy between the actual molecular weight and the observed molecular weight on SDS-PAGE is a common observation, in particular for membrane proteins, and in part reflects the effect of bound detergent [10]. However, in the control purification, no SPP signal was observed, demonstrating that the cognate peptide was able to block the isolation of SPP. Parenthetically, this method was used because it was much more effective at reducing SPP binding in the control purification than siRNA knockdown of SPP (data not shown). The identity of the co-purified proteins and their level of enrichment in the cognate-peptide-blocked control equipment were then determined by iTRAQ-labelling coupled with mass spectrometry (Figure 3A).

### iTRAQ-based analysis of the interactome of SPP

Inspection of the iTRAQ data revealed a robust enrichment of SPP (HM13) (for a list of proteins identified and their iTRAQ 117:116 ratios, see Table 1 and Additional file 1), while non-specific binders such as the frequently encountered protein HSP70, had iTRAQ 117:116 ratios for the signature mass peaks of less than 1.5 (Figure 3A). However, selective co-enrichment (117:116 ratios ≥ 1.5) of the ubiquitous 140 kDa RNA-binding protein vigilin was observed together with SPP (Figure 3B). Thirteen unique peptides were identified from vigilin in total (Figure 3C).

**Table 1:** List of proteins identified in the iTRAQ mass spectrometry analysis of SPP interacting proteins

**Protein Name**	**Accession Number**	**Symbol**	**Unique Peptides**	**Coverage (%)**	**iTRAQ Ratio (117:116)**
Signal peptide peptidase	IPI:IPI00152441.3	SPP	6	22.0	331.3
Vigilin	IPI:IPI00022228.1	VIGLN	13	14.6	4.3
10 kDa heat shock protein, mitochondrial	IPI:IPI00220362.4	HSPE1	9	72.3	1.4
Microfibrillar-associated protein 1	IPI:IPI00022790.1	MFAP1	5	20.3	1.3
Heat shock protein 60	IPI:IPI00472102.3	HSP60	23	63.8	1.2
Calnexin precursor	IPI:IPI00020984.1	CNX	9	15.5	1.1
Alpha-enolase	IPI:IPI00465248.4	ENO1	14	37.2	1.1
Glyceraldehyde-3-phosphate dehydrogenase	IPI:IPI00219018.6	GAPDH	8	33.5	1.1
Protein C14orf166	IPI:IPI00006980.1	C14orf166	7	39.8	1.1
Stress-70 protein, mitochondrial precursor	IPI:IPI00007765.5	HSPA9	43	71.4	1.0
Eukaryotic translation initiation factor 4 gamma 1	IPI:IPI00552639.2	EIF4G1	8	13.6	1.0
ATP synthase O subunit, mitochondrial precursor	IPI:IPI00007611.1	ATP5O	10	59.1	1.0
Actin, cytoplasmic 1	IPI:IPI00021439.1	ACTB	9	47.5	1.0
130 kDa leucine-rich protein	IPI:IPI00329745.4	LPPRC	11	12.8	1.0
Sideroflexin-1	IPI:IPI00009368.3	SFXN1	8	31.8	0.9
ATP-dependent DNA helicase 2 subunit 1	IPI:IPI00644712.3	Ku70	11	20.7	0.9
Prohibitin	IPI:IPI00017334.1	PHB	8	34.2	0.9
Heat shock 70 kDa protein 1	IPI:IPI00643932.1	HSP70-1	29	49.3	0.9
Splice Isoform 2 of Nucleophosmin	IPI:IPI00220740.1	NPM1	8	41.5	0.9
Protein disulfide-isomerase	IPI:IPI00010796.1	PDIA3	24	30.7	0.9
ATP synthase beta chain, mitochondrial precursor	IPI:IPI00303476.1	ATP5B	9	29.7	0.9
14-3-3 protein zeta/delta	IPI:IPI00021263.3	YWHAZ	7	45.3	0.9
NADH-ubiquinone oxidoreductase 24 kDa subunit, mitochondrial precursor	IPI:IPI00646556.1	NDUFV2	7	38.5	0.9
Heat shock 70 kDa protein 5	IPI:IPI00003362.2	HSPA5	26	52.1	0.9
ATP-dependent RNA helicase A	IPI:IPI00215638.5	DHX9	12	16.4	0.8
Splice Isoform 1 of Heat shock cognate 71 kDa protein	IPI:IPI00003865.1	HSPA8	16	51.4	0.8
ATP-dependent RNA helicase DDX3X	IPI:IPI00215637.4	DDX3X	8	15.9	0.8
Vimentin	IPI:IPI00646867.1	VIM	12	28.1	0.8
Splice Isoform A1-A of Heterogeneous nuclear ribonucleoprotein A1	IPI:IPI00465365.3	HNRNPA1	12	64.6	0.8
protein kinase C substrate 80K-H isoform 1	IPI:IPI00419384.1	PRKCSH	11	23.7	0.8
Programmed cell death 8	IPI:IPI00157908.3	PDCD8	7	14.6	0.8
peroxiredoxin 3 isoform b	IPI:IPI00374151.1	PRDX3	8	34.0	0.8
FK506-binding protein 10	IPI:IPI00303300.3	FKBP10	8	21.6	0.8
Elongation factor 2	IPI:IPI00186290.5	EEF2	12	20.3	0.8
Reticulocalbin-1	IPI:IPI00015842.1	RCN1	6	19.6	0.8
Splice Isoform 1 of Apoptosis inhibitor 5	IPI:IPI00555572.1	API5	9	24.7	0.8
Citrate synthase, mitochondrial precursor	IPI:IPI00025366.4	CS	8	19.3	0.8
Transitional endoplasmic reticulum ATPase	IPI:IPI00478540.2	VCP	8	13.9	0.8
Single-stranded DNA-binding protein, mitochondrial precursor	IPI:IPI00029744.1	SSBP1	10	74.3	0.8
Fructose-bisphosphate aldolase A	IPI:IPI00465439.4	ALDOA	6	26.7	0.8
Creatine kinase B-type	IPI:IPI00022977.1	CKB	6	23.1	0.8
Neutral alpha-glucosidase AB precursor	IPI:IPI00472068.1	GANAB	8	16.0	0.8
14-3-3 protein epsilon	IPI:IPI00000816.1	YWHAE	9	51.4	0.7
Nuclease sensitive element-binding protein 1	IPI:IPI00031812.2	NSEP1	7	48.6	0.7
Spliceosome RNA helicase BAT1	IPI:IPI00328343.7	DDX39B	9	22.2	0.7
Nucleolin	IPI:IPI00743912.1	NCL	16	31.9	0.7
ADP/ATP translocase 2	IPI:IPI00007188.4	SLC25A5	9	35.4	0.7
Prohibitin-2	IPI:IPI00027252.6	PHB2	8	43.8	0.7
ADP/ATP translocase 3	IPI:IPI00291467.6	SLC25A6	9	35.4	0.7
Splice Isoform 2 of Heterogeneous nuclear ribonucleoprotein K	IPI:IPI00216746.1	HNRPK	12	41.8	0.7
peptidylprolyl isomerase B	IPI:IPI00646304.3	PPIB	7	40.7	0.7
Peroxiredoxin-1	IPI:IPI00000874.1	PRDX1	6	34.7	0.7
Mitochondrial precursor proteins import receptor	IPI:IPI00015602.1	TOMM70A	11	26.2	0.7
Splice Isoform 1 of Polyadenylate-binding protein 1	IPI:IPI00008524.1	PABPC1	14	29.2	0.7
Splice Isoform 3 of DNA-binding protein A	IPI:IPI00219148.2	CSDA	6	31.3	0.7
Splice Isoform 3 of Interleukin enhancer-binding factor 3	IPI:IPI00414335.1	ILF3	9	16.2	0.7
Splice Isoform 2 of Probable ATP-dependent RNA helicase DDX17	IPI:IPI00651677.1	DDX17	7	19.9	0.7
40S ribosomal protein S10	IPI:IPI00008438.1	RPS10	7	49.1	0.7
Elongation factor 1-alpha 1	IPI:IPI00472724.1	EEF1A1	8	22.5	0.7
Endoplasmin	IPI:IPI00027230.3	HSP90B1	23	32.7	0.7
Dihydrolipoyllysine-residue succinyltransferase component of 2- oxoglutarate dehydrogenase complex, mitochondrial precursor	IPI:IPI00420108.4	DLSTP1	9	31.8	0.7
Splice Isoform B1 of Heterogeneous nuclear ribonucleoproteins A2/B1	IPI:IPI00396378.3	HNRNPA2B1	19	57.5	0.7
Splice Isoform Long of Splicing factor, proline- and glutamine-rich	IPI:IPI00010740.1	SFPQ	26	50.2	0.7
CKAP4 protein (Fragment)	IPI:IPI00433214.1	CKAP4	16	34.3	0.6
Calreticulin	IPI:IPI00020599.1	CALR	6	25.2	0.6
Protein disulfide-isomerase A6	IPI:IPI00644989.1	PDIA6	9	27.7	0.6
ATP synthase alpha chain, mitochondrial precursor	IPI:IPI00440493.2	ATP5A1	15	36.7	0.6
Nucleobindin-2	IPI:IPI00009123.1	NUCB2	5	23.6	0.6
40S ribosomal protein SA	IPI:IPI00553164.3	RPSA	7	39.1	0.6
Splice Isoform C1 of Heterogeneous nuclear ribonucleoproteins C1/C2	IPI:IPI00216592.1	HNRNPC	12	52.9	0.6
Heterogeneous nuclear ribonucleoprotein G	IPI:IPI00304692.1	RBMX	16	49.1	0.6
Serine hydroxymethyltransferase, mitochondrial precursor	IPI:IPI00002520.1	SHMT2	7	17.9	0.6
Protein disulfide-isomerase A4	IPI:IPI00009904.1	PDIA4	15	35.7	0.6
Proliferation-associated protein 2G4	IPI:IPI00299000.4	PA2G4	14	36.6	0.5
Non-POU domain-containing octamer-binding protein	IPI:IPI00304596.3	NONO	18	42.0	0.5
Histone H1.2	IPI:IPI00217465.4	HIST1H1C	5	34.0	0.5
Complement component 1, Q subcomponent-binding protein, mitochondrial precursor	IPI:IPI00014230.1	C1QBP	6	42.9	0.5
THO complex subunit 4	IPI:IPI00328840.7	THOC4	7	37.1	0.4

Vigilin or SPP-derived peptides were selectively enriched in the SPP purification sample compared to the control purification, while all other proteins identified had iTRAQ 117:116 ratios of less than 1.5.

### Vigilin physically interacts with SPP

**Figure 4:**
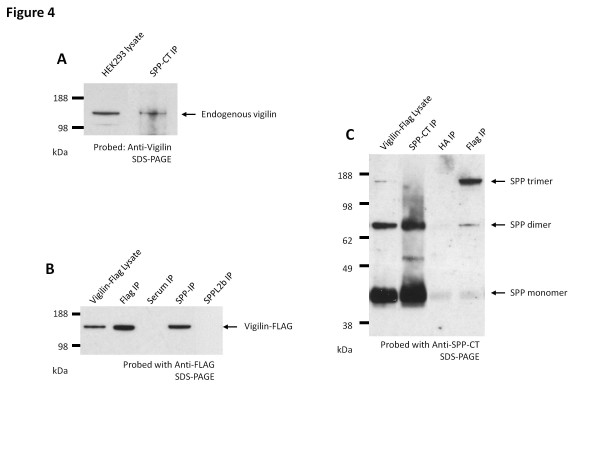
**SPP interacts with vigilin.** (**A**) Co-immunoprecipitation with anti-SPP-CT antibody pulled down endogenous vigilin. The blot was probed with anti-vigilin antibody. HEK293 cell lysate was used to align the band. (**B**) In a HEK293 cell line stably expressing FLAG-tagged vigilin, co-immunoprecipitation with anti-SPP-CT antibody pulls-down vigilin-FLAG, while antibody against SPPL2b did not pull down vigilin-Flag. The blot was probed with anti-FLAG antibody. (**C**) Co-immunoprecipitation with anti-FLAG antibody pulled-down SDS-stable SPP dimers and trimers in the FLAG-tagged vigilin expressing HEK293 cell line. The blot was probed with anti-SPP-CT antibody. Data shown are representative blots from three independent experiments.

**Figure 5:**
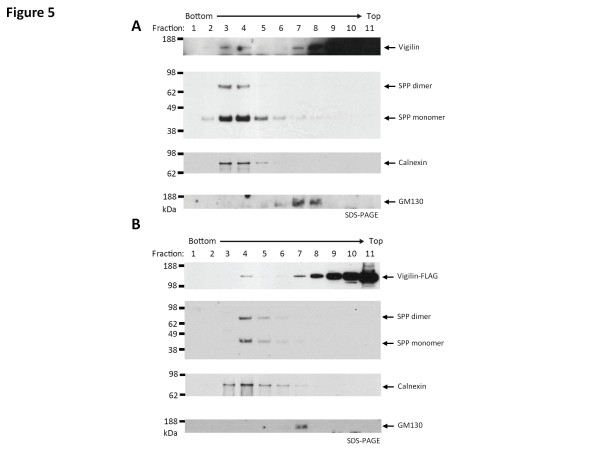
**Vigilin is found in ER fraction.** (**A**) HEK293 cells were homogenized and fractionated on an iodixanol gradient. Fractions were collected drop-wise and probed for vigilin, ER-resident SPP, ER-marker calnexin and Golgi apparatus-marker GM130. Vigilin was primarily found in the later (upper) fractions but a small proportion was present in the fractions 3 and 4, which also contained the peak fractions of SPP and calnexin. (**B**) HEK293 stably expressing vigilin-FLAG were homogenized and fractionated on an iodixanol gradient. Fractions were collected drop-wise and probed for vigilin (using anti-FLAG antibody), SPP, calnexin and GM130. Identical to the endogenous vigilin and SPP distribution, the FLAG-tagged vigilin was found mainly in the cytoplasmic fractions but also found in fraction 4, which also contained the peak fractions of SPP and calnexin.

To validate the iTRAQ results, reciprocal co-immunoprecipitation experiments from HEK293 cells were undertaken. As expected, endogenous vigilin was captured when using endogenous SPP as the bait protein (Figure 4A). The reciprocal co-immunoprecipitation experiments using anti-vigilin antibody was inconclusive because the SPP bands migrated very closely to the denatured IgG, making it difficult to differentiate SPP bands from non-specific IgG bands. In order to detect vigilin interacting with SPP after vigilin immunoprecipitation, we generated a HEK293 cell line that stably expressed FLAG-tagged vigilin. Expression pattern of FLAG-tagged vigilin in this cell line was similar to the expression pattern of the endogenous vigilin (compare Figure 5A and Figure 5B), and allowed the use of mouse anti-FLAG antibody, instead of the rabbit anti-vigilin antibody. Similar to the endogenous SPP co-immunoprecipitation experiments, the capture with anti-SPP antibody was able to specifically co-immunoprecipitate vigilin-FLAG and SPP while no bands were observed from control co-immunoprecipitations with pre-immune rabbit serum (Figure 4B). Also, this interaction is a specific because SPPL2b did not co-immunoprecipitates with vigilin, but additional SPP-like proteases will need to be tested to determine the extent of this specificity (Figure 4B). Co-immunoprecipitation experiments using anti-FLAG antibody (Figure 4C) captured vigilin-FLAG as well as its interacting partner SPP. Interestingly, the FLAG IP captured predominantly dimers and trimers of SPP but not monomers.

### Vigilin can be found in the ER fraction and co-localizes with SPP

To further explore the possibility of an *in vivo* vigilin-SPP interaction, subcellular iodixanol gradient fractionation studies were performed. In agreement with prior data showing that SPP was ER resident [11-13], SPP was found in fractions containing the ER marker calnexin, but was absent from the cytoplasmic and Golgi fractions (Figure 5A). Vigilin, as expected, was predominantly found in the cytoplasmic fractions. However, in agreement with this prior data, a small proportion of endogenous vigilin also co-fractionated in the ER membrane fractions along with SPP and calnexin. The fractions containing peak levels of the ER-bound vigilin also corresponded to the fractions containing the peak levels of SPP and calnexin. This result was not due to ‘leakage’ between the fractions or mixing during the collection of the fractions because there was a gap of 2–3 fractions where vigilin was not present in the collected fractions. Identical results were obtained for the FLAG-tagged vigilin cell line, thereby indicating that the expression of the FLAG-tagged vigilin had not caused it to be mis-localized (Figure 5B).

**Figure 6:**
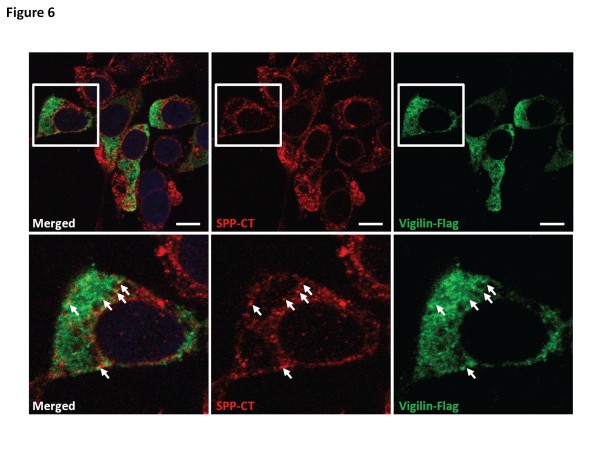
**Vigilin co-localizes with SPP.** HEK293 expressing vigilin-FLAG were immunostained with SPP-CT (red channel) and FLAG (green channel) antibodies, counter-stained with DAPI for the nuclei. *Lower panel*, magnified view of cell in white box from the *top panel*. Only a small proportion of vigilin co-localizes with SPP *in vitro* (examples of co-localization are indicated by white arrows in the magnified images). The white bar represents 10 μm.

To corroborate this biochemical evidence for an interaction, immunofluorescence studies in the HEK293 cells were performed in order to query whether co-localization of SPP and vigilin can be documented. Immunofluorescence studies were not possible with native cells expressing endogenous vigilin because the antibodies against SPP and vigilin were both raised in rabbits. Moreover, while the available anti-vigilin antibodies detect authentic vigilin-immunoreactive bands on Western blots, they also detect a few other weak non-specific bands. This raised the concern that the immunofluorescence studies could be misled by these non-specific epitopes. Consequently, we investigated the localisation of FLAG-tagged vigilin. As noted above, this strategy was safe because the biochemical fractionations studies demonstrated that neither the presence FLAG-tag nor the over-expression of the FLAG-tagged vigilin caused changes in the localisation of vigilin. In HEK293 cells expressing FLAG-tagged vigilin, the majority of the vigilin signal (green signal in Figure 6) was present in the cytoplasm, whereas the SPP signal (red signal in Figure 6) was in the ER. Nevertheless, a small proportion of SPP and vigilin showed co-localization as discrete small round foci on ER structures (yellow dots highlighted with white arrows in Figure 6). This association of vigilin with ER membranes has been previously documented [11-13].

### Vigilin is part of the 450 kDa SPP complex

**Figure 7:**
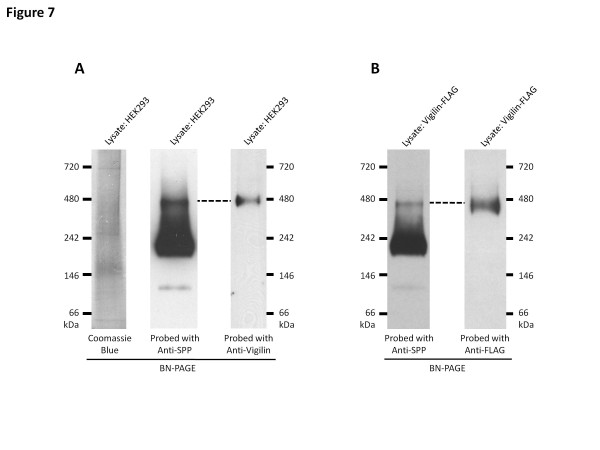
**Vigilin is part of the 450 kDa SPP complex.** (**A**) HEK293 cell lysate were solubilized in 0.5% DDM and resolved on 4-16% BN-PAGE gels. As shown on the left panel, SPP formed three distinct complexes at 450 kDa, 200 kDa and 100 kDa. Lysates probed with anti-vigilin antibody reveals that endogenous vigilin only forms one distinct complex on the BN-PAGE and that band co-migrates with the 450 kDa SPP complex. Lysates were also Coomassie stained to show that the 450 kDa SPP and vigilin containing complex was not a compression artifact of BN-PAGE gels, such as the band migrating at 700 kDa. (**B**) HEK293 vigilin-FLAG cell lysate were solubilized in 0.5% DDM and resolved on 4-16% BN-PAGE gels reveal that vigilin-FLAG also co-migrates with the 450 kDa SPP complex only. Data shown are representative blots from three independent experiments.

To determine whether vigilin was a component of any of the three SPP high molecular weight SPP complexes (450 kDa, 200 kDa and 100 kDa; Figure 1), cell lysates were resolved on BN-PAGE and probed for vigilin and SPP (Figure 7). These studies revealed that vigilin specifically co-migrates with the 450 kDa band of SPP with both native HEK293 and vigilin-FLAG lysates. Of note, the observed co-migration of SPP and vigilin in this analysis was not due to a non-specific compression of protein bands that can sometimes be observed on the BN gels (and appeared to account in this analysis for the 700 kDa band in the Coomassie-stained HEK293 lysates) (Figure 7A). Furthermore, because vigilin co-migrated only with the 450 kDa, and not with the more abundant 200 kDa and 100 kDa SPP complexes, this result argued against an artifactual interaction between vigilin and SPP. Indeed, it demonstrates that vigilin is a specific component of the high molecular weight SPP complex.

### Vigilin does not affect the aspartyl protease activity of SPP

**Figure 8:**
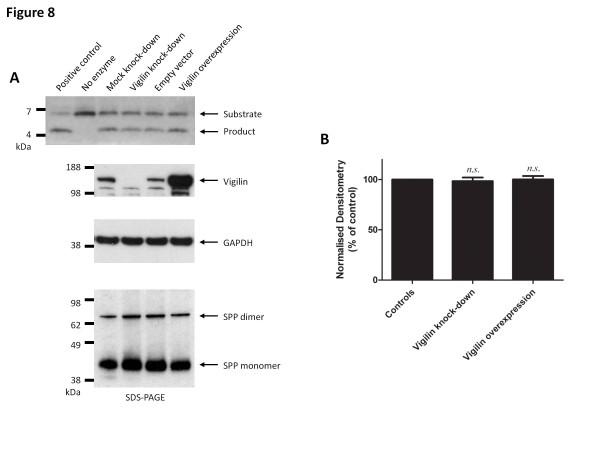
**Vigilin expression level does not alter the protease activity of SPP.** (**A**) The knockdown and over-expression of vigilin did not substantially alter the ability of SPP to cleave the prolactin substrate in the *in vitro* SPP assay. The positive control used in this study is a purified yeast homolog of SPP that cleaves the prolactin signal peptide substrate, while the negative control is set up without the addition of proteins. Expression level of vigilin was compared to the loading control, GAPDH. Total lysate concentration was adjusted to ensure equal loading of protein samples into the reaction. Data shown are representative blots from three independent experiments. (**B**) Densitometry analysis of three independent knockdown/overexpression and reactions. Bars represent mean ± s.e.m. The unpaired *t*-test was performed using Prism (*n.s.*, not significant, p-value > 0.05).

To determine if vigilin affects the proteolytic activity of SPP, we performed an *in vitro* SPP activity assay using a peptide substrate derived from the signal peptide of prolactin [14]. The SPP activity of lysates from mock-transfected HEK293 cells and cells transfected with vigilin-specific siRNAs or vigilin-FLAG expression plasmids were compared (Figure 8). No significant changes in the band intensities of the expected prolactin substrate cleavage products were observed.

## Discussion

Signal peptide peptidase (SPP), which exists as a component of several different high molecular weight membrane-bound protein complexes, has important proteolytic and non-proteolytic functions [1,5-7]. The latter functions, which are not well-characterized, may relate to protein quality control.

The biochemical and cellular data presented here suggest that the SPP-vigilin interaction is genuine and highly selective, although it is also clear that only a minor proportion of both proteins are involved in this interaction. Despite this seemingly minor interaction, vigilin molecules interact with SPP with high affinity on the basis that despite a high concentration of DDM in the solubilization step, the interaction between SPP and vigilin was still preserved. This interaction is also highly selective for SPP in high molecular weight complexes, both in immunoprecipitation and BN-PAGE experiments. There is considerable uncertainty about whether monomeric, dimeric or higher-order oligomers of SPP are catalytically active [7-9,14]. However, some evidence suggests that the higher-order species may not be catalytically active. If correct, as discussed below, this would suggest that the interaction of vigilin with these higher-order oligomers of SPP may relate to a non-proteolytic SPP activity.

A possible function of vigilin is to modulate the activity of SPP. Indeed, one obvious hypothesis is that vigilin might affect the proteolytic activity of SPP in a manner analogous to the selective modulating effect of TMP21 [15] and gSAP [16] on presenilin complex–mediated γ-secretase activity. However, unexpectedly, we found that neither vigilin knockdown nor overexpression had any effect on the proteolytic activity of SPP. It is also possible that SPP may modulate the biological activity of vigilin, although this could not be tested as no vigilin-specific biochemical assay exists.

An alternative effect of the SPP-vigilin interaction is that vigilin could modulate the putative functions of SPP in protein quality control in the ER. Testing this hypothesis will require much further work, and is beyond the focus of the current paper. However, vigilin has previously been shown to bind RNA, presumably *via* its KH domains [17-19], and can be localized with free and membrane-bound ribosomes [11-13]. The binding of mRNA by vigilin may stabilize the mRNA molecules and could be required for the localization of mRNA to actively translating ribosomes [11,20-22]. This observation, together with SPP’s putative function in binding misfolded protein, suggests that the vigilin-SPP complex could potentially be involved in the translation and quality control of membrane proteins. For instance, the vigilin-SPP interaction could ensure that SPP is near to the site of newly synthesized polypeptides destined for insertion into the ER membrane. If so, this might allow the vigilin-SPP complex to be involved in the regulation of translation and the binding/removal of newly-made misfolded membrane proteins. The latter might be achieved by a combination of: 1) dislocation/translocation back across the ER membrane (as shown for SPP’s role with MHC Class I molecules [5]); and 2) proteolytic cleavage of fragments in the membrane itself.

## Conclusions

Using an unbiased iTRAQ mass spectrometry-based approach, we have identified the multi-KH domain protein vigilin as a potential interacting protein of SPP among the 78 proteins identified. We have confirmed the interaction between SPP and vigilin using a series of experiments that included co-immunoprecipitations, subcellular fractionation, native gel and co-localization studies. Vigilin does not appear to modulate the protease activity of SPP but instead vigilin-SPP complex could have a role in membrane protein quality control in the ER.

## Methods

### Antibodies and resins

The following antibodies were used in this study: rabbit anti-SPP C-terminus polyclonal antibody (SPP-CT, Abcam, Cambridge, UK), rabbit anti-SPP N-terminus polyclonal antibody (SPP-NT, Abcam), rabbit anti-vigilin polyclonal antibody (Abcam), rabbit anti-SPPL2b polyclonal antibody (Abcam) mouse anti-FLAG monoclonal antibody (Clone M2, Sigma-Aldrich, Dorset, UK), rabbit anti-GM130 polyclonal antibody (Abcam), rabbit anti-calnexin polyclonal antibody (Abcam) and mouse anti-*myc* monoclonal antibody (Invitrogen). The HA-agarose and FLAG-agarose affinity gels were obtained from Sigma-Aldrich and Protein G-sepharose was from GE Healthcare (Little Chalfont, UK).

### Molecular cloning

Vigilin cDNA (ORFeome clone number: 100011448) was amplified by PCR using the forward (sequence: 5’-GGCGCCATGAGTTCCGTTGCAGTTTTG-3’) and reverse (sequence: 5’-GGCCGGTTACTACTTGTCATCGTCATCCTTGTAGTCTCGTTTGGGGC CCCAAGGGAG-3’) primers and inserted into the TA-cloning site of the pcDNA3.3 mammalian expression vector (Invitrogen, Paisley, UK) with a single FLAG tag (N-DYKDDDDK-C) introduced directly to the C-terminus of the vigilin cDNA.

### Cell culture, transfection and siRNA knockdown

Human Embryonic Kidney 293 (HEK293) cells were cultured in Dulbecco’s modified Eagle’s medium (DMEM, Sigma-Aldrich) containing 10% fetal bovine serum and 1% penicillin/streptomycin. Following sequence verification, the pcDNA3.3-vigilin-FLAG plasmid was transfected into HEK293 cells with FuGene 6 (Roche Applied Science, Burgess Hill, UK). Stable transfectants were clonally selected with 1 mg/ml Geneticin G418. Knock-down of SPP and Vigilin were achieved with SMARTpool siRNA (Dharmacon, Epsom, UK), which was transfected into HEK293 cells using the Dharmafect 1 reagent (Dharmacon) and, subsequently, incubated for 72–96 h.

### Membrane preparation and affinity purification of endogenous SPP

Antibodies were cross-linked to Protein G sepharose with 0.025 M Borax pH 9.45 in the presence of 20 mM dimethylpimedilate (Sigma-Aldrich) and residual uncross-linked antibody was removed with 100 mM glycine HCl pH 3.0. For the preparation of microsomal membranes, the cell pellet was resuspended and homogenized in sucrose lysis buffer (25 mM HEPES pH 7.4, 4 mM EDTA, 0.25 M sucrose, complete protease inhibitor cocktail (Roche)). Following centrifugation at 2,000 × *g* for 10 min, the supernatant was further centrifuged at 100,000 × *g* (Ti45 rotor, Beckman Coulter, High Wycombe, UK) for 60 min to isolate microsomal membranes. Membrane lysates were solubilized with 0.5% (wt/vol) n-dodecyl-β-D-maltoside (DDM, Anatrace) in HEPES buffer (25 mM HEPES pH 7.4, 150 mM NaCl, 4 mM EDTA, complete protease inhibitor cocktail). Lysates were pre-cleared with pre-immune serum cross-linked to protein G sepharose overnight, and SPP was then purified with SPP-CT antibody cross-linked to protein G sepharose. Next, the beads were subjected to at least 5 washes with HEPES buffer containing 0.02% (wt/vol) DDM and a single high-salt wash step in HEPES buffer with 300 mM NaCl. SPP and its interacting proteins were eluted by pH drop with Tris–HCl pH 3.0. To generate a negative control, anti-SPP antibody was pre-saturated with 100 μg/ml of cognate peptide (sequence: N-TESKEGTEASASKGLEKKEK-C) and washes with 100 μg/ml of the aforementioned peptide was also included.

### Protein reduction, alkylation and trypsinization

Protein-containing fractions were denatured in the presence of 9 M urea, followed by reduction with 5 mM tris-(2-carboxyethyl)-phosphine for 30 min at 60°C and alkylation with 9 mM 4-vinylpyridine for 1 h at room temperature in the dark. Samples were diluted five-fold to ensure that the concentration of urea did not exceed 2 M. Tryptic digestion was initiated by the addition of 1% (wt/wt) of side chain-modified, TPCK-treated porcine trypsin and allowed to proceed at 37°C for 6 h.

### iTRAQ labeling and mass spectrometry

The steps for the labeling of peptides with iTRAQ reagents, two-dimensional separation of peptide mixtures by offline strong-cation exchange (SCX) and online reversed phase (RP) liquid chromatography and subsequent analysis by electrospray tandem mass spectrometry have been described previously [23]. Samples derived from control and SPP-specific purifications were iTRAQ-labeled with iTRAQ-116 and iTRAQ-117 reagents, respectively.

### Database searches

Collision induced dissociation (CID) spectra were analyzed using ProteinPilot (Version 2.0, Applied Biosystems, MDS Sciex). The lists of candidate interacting proteins were subjected to the following filters: (i) all identifications of proteins had to be based on at least two CID spectra which passed the 95% confidence score returned by the ProteinPilot software; (ii) assignments to non-iTRAQ-labeled peptides or CID spectra with individual confidence scores of less than 90% were not included in the calculation of enrichment ratios based on iTRAQ signature mass signal intensities. Raw iTRAQ ratios were corrected for impurity levels of individual reagent lots determined by the manufacturer. The mass tolerance range between expected and observed masses used for database searches was ±150 ppm for MS peaks, and ±0.15 Da for MS/MS fragment ions. All samples were searched against the International Protein Index (IPI) database disseminated through EMBL/EBI.

### Co-immunoprecipitations and western blotting

Homogenized HEK293 cells were solubilized in ice-cold HEPES buffer with 0.5% DDM for 60 min and centrifuged at 100,000 × *g* for 30 min to remove insoluble material. Co-immunoprecipitations were carried out with 500–1000 μg of solubilized total lysate utilizing antibodies against SPP-CT, SPPL2b and FLAG-tag, with pre-immune rabbit serum and antibody against the HA-tag serving as the control for the SPP/SPPL2b and FLAG IPs, respectively. After pre-clearing, lysates were incubated overnight at 4°C with antibody and Protein G Sepharose or pre-conjugated affinity resins. Resin was then washed five times with 0.02% DDM in HEPES buffer. The precipitated proteins were eluted with 1 × sample buffer (LDS, Invitrogen). For immunoblotting, the samples were resolved on 12% Bis-Tris NuPAGE gels (Invitrogen), transferred onto polyvinylidene fluoride membranes (PVDF, Millipore, Watford, UK) and probed with appropriate primary and secondary antibodies.

### Blue native-polyacrylamide gel electrophoresis (BN-PAGE)

Membrane lysates solubilized in 0.5% DDM were resolved on one-dimensional BN-Page (4-16% NativePAGE Bis-Tris gels, Invitrogen), incubated in 1% SDS for 15 min and transferred onto PVDF membranes for Western Blot analysis. For the second dimension SDS-PAGE, the lanes from the BN-PAGE were excised and resolved on SDS-PAGE gels (12% Bis-Tris NuPage gels, Invitrogen).

### Subcellular fractionation on iodixanol gradients

HEK293 cells were homogenized in Tris buffer (25 mM Tris pH 7.4, 25 mM NaCl, 130 mM KCl, 1 mM EGTA, complete protease inhibitor cocktail) and centrifuged at 1,000 × *g* for 10 min then at 3,000 × *g* for 10 min. The resulting supernatant was placed on top of a step gradient consisting of 1 ml layers of 30, 25, 20, 15, 12.5, 10, 7.5, 5, and 2.5% (vol/vol) iodixanol (Sigma-Aldrich) in Tris buffer. Following centrifugation at 92,000 × *g* (SW40 rotor, Beckman Coulter) for 30 min, 11 fractions were collected from the bottom of the centrifuge tube. The fractions were analyzed for the presence of the SPP, vigilin and protein markers of the subcellular organelles.

### Immunofluorescence microscopy

HEK293 cells were plated onto cover-slips pre-coated with 0.01% (wt/vol) poly-L-lysine solution (Sigma-Aldrich). HEK293 cells were fixed with 10% (vol/vol) formalin, perforated with PBS-T (1 × PBS with 0.1% (vol/vol) Triton X-100) and blocked with 10% (vol/vol) normal goat serum (Abcam) in PBS-T. Cells were incubated for 60 min in anti-SPP and anti-vigilin primary antibodies diluted in 10% normal goat serum and subsequently incubated in the appropriate species-specific Alexa fluorescent dye conjugated secondary antibodies (Invitrogen) for 30 min. The immunostained cells were viewed by confocal microscopy (LSM510 META, Carl Zeiss, Welwyn Garden City, UK).

### In vitro SPP activity assay

The proteolytic activity of SPP was determined using an *in vitro* assay with *myc* and FLAG double tagged peptide derived from the signal peptide of prolactin [14] as the substrate. Briefly, the final SPP assay mixture (40 mM HEPES pH 7.0, 200 mM sucrose, 4 mM EDTA, 0.5 μM substrate, 10 mM β-mercaptoethanol, 0.2% (wt/vol) polar lipids extracted from *Escherichia coli* (Avanti, Alabaster, AL, USA), complete protease inhibitor cocktail and cellular lysates) was incubated on ice for 30 min to allow substrate binding and the reaction was carried out at 30°C for 2–4 h. The reaction was stopped by the addition of sample loading buffer. Substrate and product bands were separated on 16% Tricine gels (Invitrogen) and probed with anti-*myc* antibody. Reaction product and GAPDH band intensities were estimated using the ImageJ software package (version 1.45l, US National Institutes of Health, Bethesda, MD, USA) and the unpaired *t*-test was performed using the Prism statistical software package (version 5, GraphPad, La Jolla, CA, USA).

## Abbreviations

BN-PAGE = Blue native-polyacrylamide gel electrophoresis; CID = Collision-induced dissociation; DDM = N-dodecyl-β-D-maltoside; ER = Endoplasmic reticulum; gSAP = Gamma-secretase activating protein; MS = Mass spectrometry; iTRAQ = Isobaric tag for relative and absolute quantitation; SPP = Signal peptide peptidase.

## Competing interests

The author(s) declare that they have no competing interests.

## Authors’ contributions

PStGH and GSU conceived the study and participated in its design and coordination. SHJL, AHWJ, GSU, and WM performed the experiment. All authors analysed the data, prepared and approved the final manuscript.

## Supplementary Material

Additional file 1 **Complete list of peptides identified in the iTRAQ mass spectrometry analysis of SPP interacting proteins.** Vigilin or SPP-derived peptides were selectively enriched in the SPP purification sample compared to the control purification, while all other proteins identified had iTRAQ 117:116 ratios of less than 1.5. Click here for file
